# Expression of Toll-Like Receptors (TLR2 and TLR4) in the Eyes of Mice with Disseminated Acanthamoebiasis

**DOI:** 10.1155/2019/1401894

**Published:** 2019-06-12

**Authors:** Karolina Kot, Danuta Kosik-Bogacka, Natalia Łanocha-Arendarczyk, Agnieszka Wojtkowiak-Giera, Agnieszka Kolasa-Wołosiuk

**Affiliations:** ^1^Department of Biology and Medical Parasitology, Pomeranian Medical University in Szczecin, 70-204 Szczecin, Poland; ^2^Independent of Pharmaceutical Botany, Department of Biology and Medical Parasitology, Pomeranian Medical University in Szczecin, 70-204 Szczecin, Poland; ^3^Department of Biology and Medical Parasitology, Poznan University of Medical Sciences, Fredry 10, 61-701 Poznan, Poland; ^4^Department of Histology and Embryology, Pomeranian Medical University in Szczecin, 70-204 Szczecin, Poland

## Abstract

Toll-like receptors (TLRs) play a key role in the innate immune response to numerous pathogens, including* Acanthamoeba* spp. The aim of this study was to determine the expression of TLR2 and TLR4 in the eyes of mice following intranasal infection with* Acanthamoeba* spp. in relation to the host's immunological status. Amoebae used in this study were isolated from the bronchial aspirate of a patient with acute myeloid leukemia (AML) and atypical symptoms of pneumonia. We found statistically significant differences in the expression of TLR2 and TLR4 in the eye of immunocompetent mice at 8, 16, and 24 days after* Acanthamoeba* spp. infection (dpi) compared to control group. Immunosuppressed mice showed significant differences in the expression of TLR2 at 16 and 24 dpi compared to uninfected animals. Our results indicate that TLR2 and TLR4 are upregulated in the eyes of mice in response to* Acanthamoeba* spp. We suggest that it is possible for trophozoites to migrate through the optic nerve from the brain to the eyes. The course of disseminated acanthamoebiasis may be influenced by the host's immunological status, and the observed changes in expression of TLR2 and TLR4 in the host's organs may indicate the role of these receptors in the pathomechanism of acanthamoebiasis.

## 1. Introduction

Parasitic infections can initiate both specific and nonspecific immune responses. Toll-like receptors (TLRs) are a family of membrane receptors that play a key role in the nonspecific immune response, recognizing pathogen-associated molecular patterns (PAMPs) common to most pathogenic microorganisms [1]. Recognition and binding of PAMPs lead to the dimerization or oligomerization of TLRs and recruitment of intracellular signaling molecules. Binding of molecules to TLR leads to the activation of MAP kinases, including extracellular signal-regulated kinases ERK1/2 and transcription factor NF-*κ*B, which controls the expression of genes encoding proinflammatory cytokines, such as TNF-*α* and IL-2 [2]. It also leads to the activation of interferon regulatory factor 3 (IRF3), which regulates the expression of type I (I IFN) interferons, mainly IFN-*α* [2].

*Acanthamoeba* spp. can be found in a wide variety of environments: soil, dust, air, natural water, tap water, drinking and bottled water, seawater, swimming pools, sewage, sediments, air-conditioning units, dental treatment units, hospitals and dialysis units, and contact lenses. They also often infect bacterial, yeast, and mammalian cell cultures [3]. In earlier studies, antibodies for* Acanthamoeba* antigens were found in 50% to 100% of studied populations, indicating that contact with these pathogens is common [4, 5].* Acanthamoeba* are the causative agents of granulomatous amoebic encephalitis (GAE), a fatal disease of the central nervous system (CNS), and amoebic keratitis (AK), a painful vision-threatening disease of the eyes [6, 7]. Several species of* Acanthamoeba*, including* A. castellanii, A. polyphaga, A. hatchetti, A. culbertsoni, A. rhysodes, A. griffini, A. quina*, and* A. lugdunensis*, have been reported to cause AK [8]. AK is most common in contact lens wearers (>8%), occurring when lenses are stored contrary to the recommendations of doctors and manufacturers, as well as in patients with mechanical corneal damage [9, 10]. Studies of* Acanthamoeba* keratitis found that the host immune system prevents the spread of amoebae into the eye and other organs through the recruitment of neutrophils [11–13]. However, there is some data showing infection of the eye in hosts with disseminated acanthamoebiasis [14].

The receptor responsible for immune recognition of* Acanthamoeba* spp. is TRL4 [15]. Based on* in vitro* and* in vivo* studies, it was found that TLR4 is expressed during amoebic infection in AK. Activation of TLR4 stimulates the pathways TLR4-MyD88-NF-*κ*B and TLR4-ERK1/2 to induce the secretion of inflammatory cytokines, including CXCL2, IL-8, TNF-*α*, and IFN-*α* [15–17]. TLR activation also plays a significant role in directing T helper cell (Th) differentiation. The presence of TLR ligands mainly initiates a Th1 response but may also lead to the appearance of induced regulatory T lymphocytes [18]. Recent studies on the AK mouse model showed that amoebic infection induces Th17 lymphocytes and Treg lymphocytes in the cornea [19]. In our earlier studies on disseminated acanthamoebiasis, induction of Th1, Th2, and Th17 expression was seen in immunocompetent hosts in the late stages of* Acanthamoeba* spp. infection, whereas, in hosts with suppressed immunity, we observed a strong Th1 response, without Th17 [20].

Previous studies on the role of TLRs in acanthamoebiasis have focused mainly on the expression or activation of TLRs in the corneas of people with AK, after contact lenses with clinical isolates of* Acanthamoeba* were placed onto the center of corneas or the* Acanthamoeba* solution was applied to previously scratched corneas [15, 17]. No studies to date have addressed the implications of the role of TLR2 and TLR4 in* Acanthamoeba* spp. eye infections in intranasally infected immunocompetent and immunosuppressed hosts with disseminated acanthamoebiasis. Our previous study showed that AM22 amoeba strain caused changes in the activity of cyclooxygenase 1 and 2 (COX-1 and COX-2) and antioxidant enzymes in the lungs of mice as well as changes in the activity of matrix metalloproteinases (MMP-2, MMP-9) and their tissue inhibitors (TIMP-1, TIMP-3) in the cerebral cortex and hippocampus of mice [21–23]. The purpose of this study was to determine whether* Acanthamoeba* spp. may affect the expression of TLR2 and TLR4 in eye, in relation to the host's immunological status.

## 2. Materials and Methods

### 2.1. Animal Model

Our experimental animal model has been described in our previous research [21, 24]. Adult male, Balb/c mice (~23 g, 6-10 months, Center of Experimental Medicine, Medical University in Białystok, Poland) were housed individually on a 12 h:12h light/dark cycle under controlled temperature with free access to food and water.

All animal procedures were carried out in accordance with established practices for laboratory animal work according to the “Guide for the Care and Use of Laboratory Animals.”

**Figure 1:**
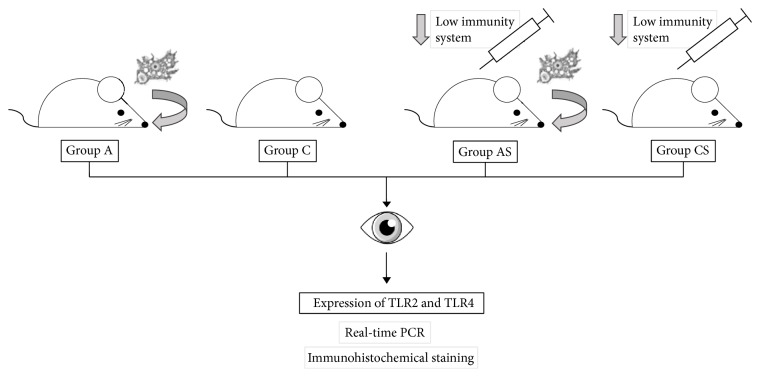
Schematic diagram of conducted experiment (group C: uninfected immunocompetent mice; group CS: uninfected mice treated with an immunosuppressive drug; group A:* Acanthamoeba* spp. infected immunocompetent mice; group AS:* Acanthamoeba* spp. infected mice treated with an immunosuppressive drug).

The mice (n=96) were divided into four groups (Figure 1):

- Group C: uninfected immunocompetent mice (control group; n=18)
- Group CS: uninfected mice treated with an immunosuppressive drug (n=18)
- Group A:* Acanthamoeba* spp. infected immunocompetent mice (n=30)
- Group AS:* Acanthamoeba* spp. infected mice treated with an immunosuppressive drug (n=30)

Mice from groups A and AS were infected by intranasal inoculation with 3 *μ*l of suspension containing 1-2x10^4^ of* Acanthamoeba* spp. strain AM22 isolated from a patient with acute myeloid leukemia (AML) and atypical pneumonia [25]. Cultures were incubated at 37°C in NN Agar covered with a suspension of deactivated* Escherichia coli* according to standard protocol [26]. The amoebae were rinsed from the agar surface with saline (0.9% NaCl) and then centrifuged at 3000 x g for 10 minutes. Centrifuged amoebae were counted in the Bürker chamber and used for the experiment. Animals from control groups (C and CS) were given the same volume of saline (3 *μ*l 0.9% NaCl). To suppress immunity, animals from AS and CS groups were intraperitoneally given (i.p.) 0.22 mg (10 mg/kg body weight) methylprednisolone sodium succinate (MPS, Solu-Medrol, Pfizer, Europe MA EEIG) dissolved in 0.1 mL 0.9% saline daily for four days before inoculation with* Acanthamoeba* spp. [21, 27].

Mice were sacrificed by sodium pentobarbital (Euthasol vet, FATRO) injection administered intraperitoneally at 2 mL/kg body weight. The mice were weighed, and then eyes were removed for analysis. The virulence of* Acanthamoeba* spp. was determined by the degree of infection. Eyes were placed on NN agar and incubated at 41°C to assess infection intensity [28].

The study was approved by the Local Ethical Committee for Experiments on Animals (No. 29/2015 and 64/2016).

### 2.2. Isolation of RNA and Conversion of cDNA by Reverse Transcription

The expression of TLR2 and TLR4 genes at the mRNA level in the eyes of mice from all groups was examined using reverse transcription polymerase chain reaction (RT-PCR). Tissues were homogenized in liquid nitrogen, and total RNA was isolated according to the manufacturer's instructions (Qiagen, Germany). One microgram of RNA from segments of eye was reverse transcribed with oligo (dT) primer in a 20 *μ*l reaction (first-strand cDNA synthesis using M-MLV RT Kit; Invitrogen, California) to obtain cDNA. Successful cDNA conversions were confirmed by amplification using conventional PCR (GeneAmp PCR System 2400, Applied Biosystems).

### 2.3. Real-Time PCR

TLR2 and TLR4 gene expressions in the eyes were measured by quantitative real-time polymerase chain reaction (Q-PCR). Q-PCR was carried out in a LightCycler real-time PCR detection system from Roche Diagnostic GmbH (Mannheim, Germany) using SYBR Green I as detection dye and target cDNA was quantified by relative quantification using a calibrator. The calibrator was prepared as a cDNA mix from all samples. The housekeeping gene PBGD was amplified as the reference gene for mRNA quantification. The quantity of TLR2 and TLR4 transcripts in each sample was standardized by the geometric mean of PBGD transcript level; more analytical procedures are given by Wojtkowiak-Giera et al. [29]. The amounts of TLR2 and TLR4 mRNA are expressed as the multiplicity of these cDNA concentrations in the calibrator.

### 2.4. Immunohistochemical Staining

Samples fixed in 4% buffered formalin solution (Avantor, Poland) were subsequently embedded in paraffin and cut into sections of 4 *μ*m thickness. The sectioned tissue was deparaffinized in microwave and irradiated with citrate buffer (pH 6.0) to induce epitope retrieval. After slow cooling to room temperature, slides were washed in PBS twice for 5 min and then incubated with primary antibodies overnight (4°C). Immunohistochemistry was performed using specific primary rabbit polyclonal antibodies against TLR2 and TLR4 (Santa Cruz Biotechnology, Inc., cat. no. sc-10739 and sc-30002, respectively) in a final 1:500 dilution. Sections were stained with an avidin-biotin-peroxidase system with diaminobenzidine as the chromogen (DakoCytomation, Code K0679), performed according to staining procedure instructions included. Sections were washed in distilled H_2_O and counterstained with hematoxylin. For a negative control, specimens were processed in the absence of primary antibodies. Positive staining was defined by visual identification of brown pigmentation using light microscope (Leica, DM5000B, Germany).

### 2.5. Statistical Analysis

Statistical analysis was performed using StatSoft Statistica 10.0 and Microsoft Excel 2016. Intergroup comparisons were performed using Mann-Whitney U test. The significance level was* p*<0.05.

## 3. Results

### 3.1. Differences in Expression of tlr2 and tlr4 Genes in the Eyes of Immunocompetent and Immunosuppressed Mice

There were significant differences in the levels of mRNA expression of TLR2 and TLR4 in immunocompetent* Acanthamoeba* spp. infected mice (group A) at 8, 16, and 24 dpi, and in the expression of TLR2 in immunosuppressed* Acanthamoeba* spp. infected mice (AS) between 16 and 24 dpi.

**Figure 2:**
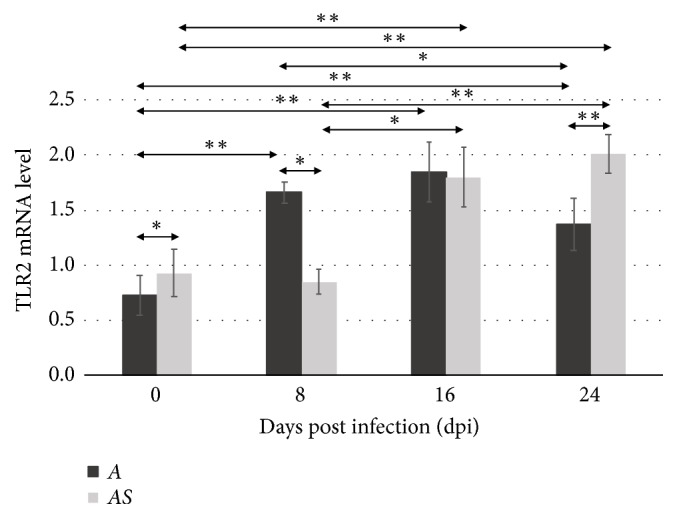
The mRNA expression of the* tlr2* gene in the eye of uninfected (0 dpi) and infected mice at 8, 16, and 24 days after* Acanthamoeba* spp. infection (dpi), according to the immunological status of hosts (A: immunocompetent mice; AS: immunosuppressed mice). The data represent mean ± standard deviation (SD) for eight independent experiments; *∗p*<0.05, *∗∗p*<0.01, compared with control value from uninfected mice (Mann–Whitney U test).

In immunocompetent* Acanthamoeba* spp. infected mice (A), TLR2 mRNA expression significantly increased at 8, 16, and 24 dpi compared to the uninfected mice (C) (*p*<0.01, Figure 2). At 24 days after* Acanthamoeba *spp. infection, TLR2 mRNA expression decreased compared to 8 dpi (*p*<0.05). In* Acanthamoeba* spp. infected mice treated with the immunosuppressive drug (AS), TLR2 mRNA expression increased significantly at 24 dpi (*p*<0.01). We found statistically significant differences in the expression of TLR2 between the uninfected immunocompetent mice (group C) and those observed at 16 (*p*<0.05) and 24 (*p*<0.01) days after* Acanthamoeba* spp. infection (group AS). Moreover, statistically significant differences in expression were observed between immunocompetent (A) and immunosuppressed (AS) mice at 0 (*p*<0.05), 8 (*p*<0.05), and 24 (*p*<0.01) days after* Acanthamoeba* spp. infection.

**Figure 3:**
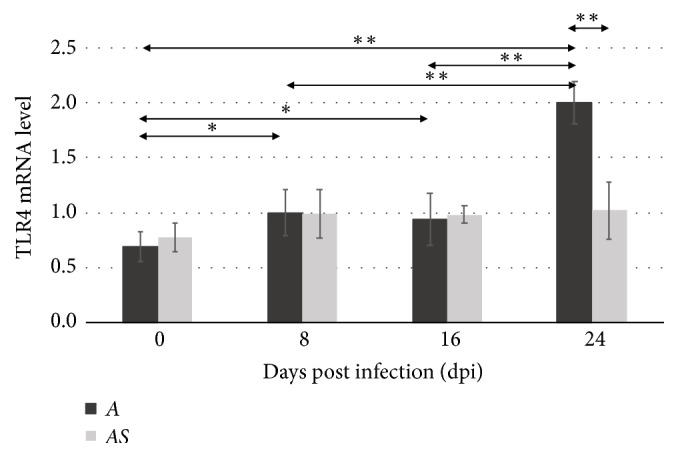
The mRNA expression of the* tlr4* gene in the eye of uninfected (0 dpi) and infected mice at 8, 16, and 24 days after* Acanthamoeba* spp. infection (dpi), according to the immunological status of hosts (A: immunocompetent mice; AS: immunosuppressed mice). Data represent mean ± standard deviation (SD) for eight independent experiments; *∗p*<0.05, *∗∗p*<0.01, compared with control value from uninfected mice (Mann–Whitney U test).

The expression of TLR4 in immunocompetent* Acanthamoeba* spp. infected mice (A) was increased at 8 (*p*<0.05), 16 (*p*<0.05), and 24 (*p*<0.01) dpi compared to uninfected* Acanthamoeba* spp. infected mice (C) (Figure 3). Statistically significant differences were observed in the immunocompetent* Acanthamoeba* spp. infected mice (A) between 8 and 24 dpi, and between 16 and 24 dpi (*p*<0.01). In mice treated with the immunosuppressive drug, no statistically significant differences were found in the level of TLR4 expression between uninfected mice (CS) and those infected with* Acanthamoeba* spp. (AS). Comparison of TLR4 expression levels between immunocompetent (A) and immunosuppressed (AS) mice showed a statistically significant difference only at 24 days after* Acanthamoeba* spp. infection (*p*<0.01).

### 3.2. TLR2 and TLR4 Immunoexpression Immunohistochemistry Reaction in the Eyes of Immunocompetent and Immunosuppressed Mice

**Figure 4:**
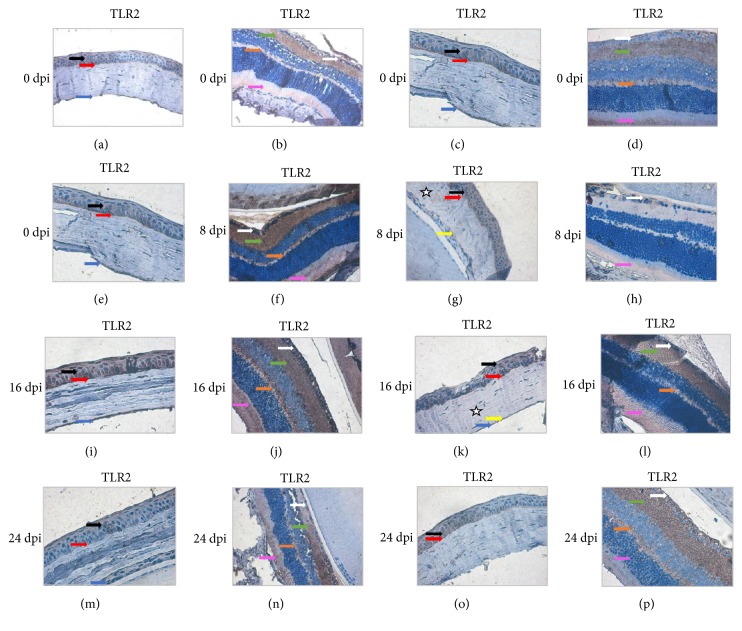
Immunohistochemical staining with primary anti-TLR2 antibodies in the corneas (a, c, e, g, i, k, m, o) and retinas (b, d, f, h, j, l, n, (p)) of immunocompetent (a, b, e, f, i, j, m, n) and immunosuppressed mice (c, d, g, h, k, l, o, p) from control groups (0 dpi) and at 8, 16, and 24 days after* Acanthamoeba* spp. infection (dpi). Magnification x40 (black arrows: corneal epithelium; red arrows: external limiting membrane, Bowman's membrane; white stars: stroma of cornea, substantia basalis; yellow arrows: Descemet's membrane; blue arrows: corneal endothelium; white arrows: optic nerve fiber layer; green arrows: inner plexiform layer; orange arrows: outer plexiform layer; pink arrows: layer of rods and cones).

In uninfected immunocompetent mice (C), immunohistochemical detection of TLR2 was mainly localized to the corneal epithelium (Figure 4(a), black arrows), Bowman's membrane (Figure 4(a), red arrows), and corneal endothelium (Figure 4(a), blue arrows). TLR2 expression in* Acanthamoeba* spp. infected immunocompetent mice (A) was detected in the same structures as in the control group (C). The highest expression was observed at 8 dpi (Figure 4(e)).

Brown pigmentation indicating TLR2 immunohistochemical staining was observed in the corneal epithelium (black arrows), Bowman's membrane (red arrows), corneal stroma (white asterisks), and corneal endothelium (blue arrows). The level of expression of TRL2 in uninfected immunosuppressed mice (CS) was comparable to that of uninfected immunocompetent control mice (C). In* Acanthamoeba* spp. infected immunosuppressed mice (AS), TLR2 was expressed in all corneal layers, with the highest expression levels observed at 16 dpi. In immunosuppressed* Acanthamoeba* spp. infected mice (AS) at 8 and 24 dpi, the corneal endothelium (Figure 4(g) and Figure 4(o)) and Descemet's membrane (Figure 4(o)) were found as TLR2-negative at 24 dpi.

In the retinas of immunocompetent (A) and immunosuppressed (AS)* Acanthamoeba* spp. infected mice, TLR2 expression was observed in the optic nerve fiber layer (white arrows), the inner and outer plexiform layers (green and orange arrows, respectively), and the rods and cones layer (pink arrow) (Figure 4). TLR2 immunoexpression was most intense at 8 and 16 days after* Acanthamoeba* spp. infection (Figures 4(f) and 4(j)). Changes in immunoexpression of TLR2 were also observed in the visual part of the retina in immunosuppressed* Acanthamoeba* spp. infected mice (AS). The highest TLR2 expression levels were observed at 24 dpi (Figure 4(p)).

**Figure 5:**
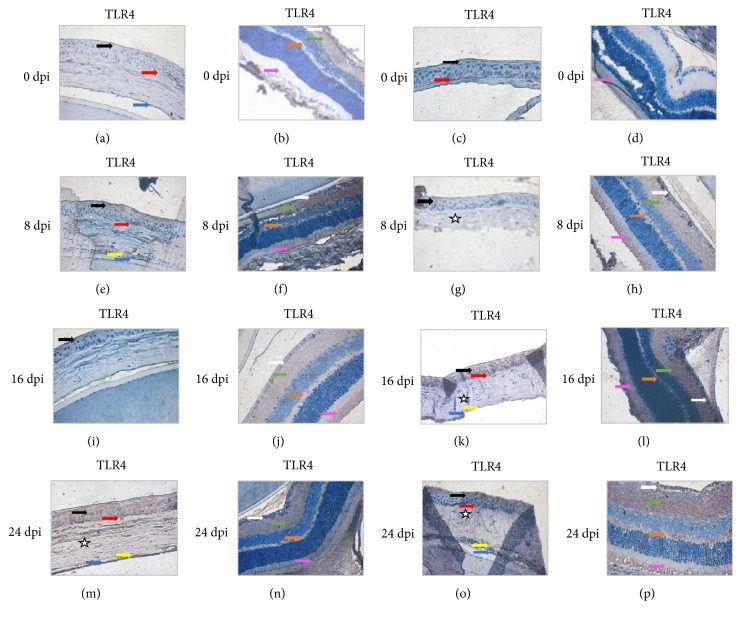
Immunohistochemical staining with primary anti-TLR4 antibodies in the corneas (a, c, e, g, i, k, m, o) and retinas (b, d, f, h, j, l, n, (p)) of immunocompetent (a, b, e, f, i, j, m, n) and immunosuppressed mice (c, d, g, h, k, l, o, p) from control groups (0 dpi) and at 8, 16, and 24 days after* Acanthamoeba* spp. infection (dpi). Magnification x40 (black arrows: corneal epithelium; red arrows: external limiting membrane, Bowman's membrane; white stars: stroma of cornea, substantia basalis; yellow arrows: Descemet's membrane; blue arrows: corneal endothelium; white arrows: optic nerve fiber layer; green arrows: inner plexiform layer; orange arrows: outer plexiform layer; pink arrows: layer of rods and cones).

Expression of TLR4 in the corneas of uninfected immunocompetent mice (C) was similar to that of TLR2. Similarly, the highest TLR4 expression levels were seen at 24 dpi (Figure 5). TLR4 expression at 24 dpi was observed in all corneal layers, including the corneal epithelium (black arrow), Bowman's membrane (red arrow), corneal stroma (white star), posterior limiting (Descemet's) membrane (yellow arrow), and corneal endothelium (blue arrow). In uninfected immunosuppressed mice (CS), the weakest expression of TLR4 was observed at 8 dpi (Figure 5(g)) and the strongest at 16 dpi (Figure 5(k)) and 24 days after amoeba infection (Figure 5(o)). In the cornea, expression of both TLR2 and TLR4 was found only in the perinuclear space.

In the visual part of the retina in the eyes of immunocompetent (C) and compromised mice (CS), expression of TLR4 was observed in the optic nerve fiber layer (white arrow), the inner plexiform layer (green arrow), the outer plexiform layer (orange arrow), and rods and cones layer (pink arrow) (Figure 5). In immunocompetent hosts, the intensity of the immunohistochemical reaction increased 8 days after* Acanthamoeba* spp. infection (Figure 5(f)), then fell at 16 dpi (Figure 5(j)), and increased again, reaching a maximum at 24 dpi (Figure 5(n)). Brown pigmentation indicating immunohistochemical detection of TLR4 in the visual part of the retina of immunosuppressed animals (CS or AS) was different than that observed for TLR2. The weakest expression of TLR4 was observed in the immunocompetent control group (C) and was visible only in the layer of rods and cones (Figure 5(d)). The intensity of TLR4 expression increased with the duration of infection (Figures 5(h), 5(l), and 5(p)). No TLR2-positive or TLR4-positive cells were found in the ganglion cell layer or in the inner or outer nuclear layers in either immunocompetent or immunosuppressed mice.

## 4. Discussion

Many authors have reported an important role for TLRs in protective immunity against* Acanthamoeba* infection. For instance, in a study where mice were infected with* Acanthamoeba* spp. isolated from a patient with* Acanthamoeba* keratitis and from environmental water samples, TLR2 and TLR4 showed increased expression in pneumocytes, interstitial cells, and epithelial cells of the bronchial tree [30]. Increased TLR2 and TLR4 expression was also observed in neurons, glial cells, and endothelial cells within the neocortex [29]. Derda et al. [30] observed that, in the lungs of mice infected with* Acanthamoeba* spp., the expression of TLR2 was higher than the expression of TLR4. Moreover, the authors observed increased expression of TLR2 and TLR4 from 2 to 30 days after* Acanthamoeba* spp. infection.

Experimental models of amoebic corneal inflammation are created by introducing trophozoites through intracorneal or intraconjunctival injection, deposition into the conjunctival cul-de-sac, or topical application to an abraded corneal surface [31]. In human and laboratory animals corneal epithelial cell lines, it was found that TLR4 plays a major role in corneal inflammation caused by* Acanthamoeba* spp. infection [15–17].* In vitro*, immunological interactions between this amoeba and the corneal epithelium and corneal stroma were also found to increase expression of TLR2 [16]. However, on repeating the experiment in the rat model, no increase in TLR2 expression at either the mRNA or protein level was found [17]. In scientific literature, there is lack of data on the expression of TLRs in the eyes after intranasal amoeba inoculation conditioning disseminated acanthamoebiasis. In the present study, more statistically significant differences in mRNA level expression were observed for TLR2 than TLR4 in the eye of mice inoculated with* Acanthamoeba*. Johnson et al. [32] demonstrated previously that the activation of TLR2 by certain ligands induced neutrophil recruitment and increased corneal thickness. Our histological examination of the cornea did not reveal inflammatory cells in studied mice; however, we observed an increased corneal thickness due to an increase in the number of layers of nonkeratinized stratified squamous epithelium [unpublished].

The level of mRNA expression of both TLR2 and TLR4 in immunocompetent mice was significantly higher at 8, 16, and 24 days after* Acanthamoeba* spp. infection compared to the control group. In immunocompromised mice, statistically significant differences in TLR2 expression compared to uninfected animals were seen at 16 and 24 dpi. In terms of TLR4 receptor expression, in mice immunosuppressed by methylprednisolone no statistically significant differences were found in comparison to 0 dpi. Jin et al. [33] showed that hydrocortisone increased expression of TLR2 and TLR4 in human corneal epithelial cell lines (HCEC), while in human corneal fibroblasts (HCF), expression of these receptors was decreased. The authors suggest that topical treatment with steroids may promote opportunistic corneal infections by inhibiting the release of the innate immune response mediators through the interaction of TLR with this glucocorticosteroid. Hara et al. [34] showed that dexamethasone can increase HCEC susceptibility to viral infections by altering signaling pathways of Toll-like receptors. In our study, mice were immunosuppressed by administration of the glucocorticosteroid methylprednisolone. Statistically significant differences were observed between the expressions of TLR2 and TLR4 in immunocompetent and immunosuppressed mice. TLR2 expression was higher in the AS group compared to the A group and increased with 24 days after* Acanthamoeba* spp. infection. Regarding TLR4, the expression of this receptor in immunosuppressed mice was reduced at 24 dpi compared to the immunocompetent group.

Differences between the levels of expression observed in this paper and those of other authors' papers may result from differences in the method of infection, type of immunosuppressant, our performance of analyses in all structures of the eye, and/or differences between* Acanthamoeba* strains used.

Injection of* Acanthamoeba* spp. trophozoites into the anterior chamber of the eye did not cause morphological changes in the retina or posterior chamber of the eye [35]. However, in another study, an injection of* Acanthamoeba* into the corneal stroma led to severe encephalitis in some animals [36]. This is explained by the belief that amoebae can migrate along corneal nerves [37]. Chandra et al. [14] described the case of an immunocompetent patient diagnosed with meningitis caused by* Acanthamoeba* spp., in whom examination of the posterior part of the eye revealed subarachnoid inflammation surrounding the optic nerve. Moreover, trophozoites of* Acanthamoeba* spp. were found in the perioral space and optic nerve. In this study,* Acanthamoeba* spp. were reisolated from the eye and optic nerves of mice infected with amoebae.

Confocal microscopy and histological analysis of the cornea in established cases of* Acanthamoeba* keratitis show corneal oedema, presence of inflammatory cells in the corneal stroma, trophozoites and cysts of* Acanthamoeba* spp. in all intraepithelial and stromal layers of the cornea, as well as regional stromal necrosis [17, 36, 38]. In a patient with* Acanthamoeba* keratitis, Kato et al. [39] observed polymorphonuclear leukocytes in the corneal stroma, an abscess in the granulation tissue at the sclera near the ciliary body, and macrophages and lymphocytes surrounding blood vessels. There was no inflammation of the retina and vascular system. In our study, despite the fact that* Acanthamoeba* spp. were administered intranasally, we found morphological changes in the eye of immunosuppressed mice, including invagination of Bowman's membrane into the substantia propria of corneal stroma and an increase in the number of layers of nonkeratinized stratified squamous epithelium. Moreover, we found morphological changes of the ciliary body and dilatation of nuclear layers of retina. However, no inflammatory cells or amoeba developmental forms were found in eye structures [unpublished].

Based on the study conducted on this murine model, it was found that, through changes in the activity of biochemical indexes and amoeba reisolation from fragments of the lungs, kidneys, and liver after intranasal amoeba inoculation, the animals developed disseminated acanthamoebiasis [21, 22, 24]. Moreover, the presence of* Acanthamoeba* spp. was also observed in the cerebral cortex and hippocampus of mice [23]. On the basis of previous research and data presented in this study, we imply that* Acanthamoeba* spp. are capable of migrating through the optic nerve from the brain to the eyes. Therefore, future research should investigate the possibility of amoeba migration through nerves from the eyes to the brain, at the same time examining whether patients with AK may develop disseminated acanthamoebiasis.

## 5. Conclusions

The present study indicates that TLR2 and TLR4 are upregulated in the eyes of mice in response to* Acanthamoeba* sp. infection. The observed changes in the expression of both toll-like receptors may confirm involvement of the innate immune system in the pathomechanism of intranasally triggered acanthamoebiasis. The results suggest that it may be possible for trophozoites to migrate through the optic nerve from the brain to the eyeball.

## Data Availability

The data used to support the findings of this study are available from the corresponding author upon request.
